# Saponin Molecules from Quinoa Residues: Exploring Their Surfactant, Emulsifying, and Detergent Properties

**DOI:** 10.3390/molecules29204928

**Published:** 2024-10-18

**Authors:** Kiara A. García Bustos, Salvador Sanchez Muñoz, Silvio S. da Silva, Miguel A. D. Flores Alarcon, Júlio C. dos Santos, Gilberto J. Colina Andrade, Ruly Terán Hilares

**Affiliations:** 1 Laboratorio de Bioprocesos, Facultad de Ciencias Farmacéuticas, Bioquímicas y Biotecnológicas, Universidad Católica de Santa María—UCSM, Urb. San José s/n—Umacollo, Arequipa 04000, Peru; kgarcia@ucsm.edu.pe (K.A.G.B.); gcolina@ucsm.edu.pe (G.J.C.A.); 2 Department of Biotechnology, Engineering School of Lorena, University of São Paulo (EEL-USP), Lorena 12602-810, SP, Brazil; salvador.sanchez@usp.br (S.S.M.); silviosilverio@usp.br (S.S.d.S.); miguel.floresalarcon@usp.br (M.A.D.F.A.); jsant200@usp.br (J.C.d.S.)

**Keywords:** saponin, *Chenopodium quinoa*, surfactant properties, circular economy

## Abstract

The indiscriminate use of synthetic surfactants, despite their desirable properties, poses significant environmental risks to ecosystems. This study explores saponins extracted from quinoa (*Chenopodium quinoa*) residues as a sustainable alternative. Saponin extract (SE) with 42% purity, obtained through hydrodynamic cavitation and membrane technology, was analyzed to determine its techno-functional properties. The critical micelle concentration (CMC) was 1.2 g/L, reducing the surface tension (ST) from 72.0 mN/m to 50.0 mN/m. The effects of temperature (30–90 °C), pH (2–12), and salinity (10,000–150,000 ppm NaCl) on ST and the emulsification index (EI) were assessed using a Box–Behnken design. Optimized conditions yielded an ST of 49.02 mN/m and an EI of 63%. Given these characteristics, SE was evaluated as a detergent across diverse swatches. This study showcases the attributes of quinoa-derived saponins, highlighting their potential for eco-friendly detergent applications.

## 1. Introduction

Surfactants stand as one of the most pressing emerging contaminants, persistently released into the environment via wastewater treatment plants or directly discharged in watercourses, with this last problem particularly prevalent in underdeveloped regions [1,2]. These molecules possess both hydrophilic (polar charged or uncharged head group) and hydrophobic (non-polar hydrocarbon tail) regions, making them amphipathic and capable of reducing interfacial tension and stabilizing foams or emulsions [3,4]. Most surfactants are petroleum-based and chemically synthesized, serving various domestic and industrial purposes such as emulsification, foaming, detergency, wetting, dispersing, or solubilization [5,6,7,8]. However, the persistence of the transformed products in an environment is of great concern to environmental sustainability and healthy ecosystems [9,10,11,12,13].

The global market size of surfactants is currently about USD 42.1 billion, and it is projected to reach USD 52.4 billion by 2025 [13]. An increment in their production involves an increase in their environmental impact, and as a result, there is a high demand for the development of new surfactants based on natural products such as plant extracts [14].

Biosurfactants are amphiphilic biological compounds produced by a variety of microorganisms [15] and plants that can be obtained from various raw materials, including sugars, oils, and wastes, exhibiting tensioactive and emulsifying properties [16]. Patel et al. [17] found that utilizing renewable resources for surfactant synthesis instead of petrochemicals could reduce CO_2_ emissions by 37% in the European Union. Moreover, biosurfactants present other advantages, like their high biodegradability, low toxicity, thermal stability, resistance to extreme values of pH and ionic strength, and solubility in an alkaline medium [16,18].

One of the most extensively researched sources of surfactants is plants containing saponins. Saponins, natural surface-active molecules, are found in various plant extracts, some of which exhibit high interfacial activity [14]. These natural surfactants consist of hydrophobic aglycones and hydrophilic sugar residues, giving them a heterogeneous amphiphilic structure [19]. *Chenopodium quinoa*, a valuable food source gaining prominence worldwide, contains diverse bitter-tasting saponins, serving as significant antinutritional factors [20].

Quinoa grains are typically subjected to scarification, a mechanical abrasion process that removes the outer layer and results in a yellowish powder, which constitutes approximately 5–8% (*w*/*w*) of the grain. This powder is rich in fiber, phenolic compounds, and saponins. Currently, this bitter residue is often discarded without treatment, leading to environmental pollution due to its high saponin content (exceeding 15% *w*/*w*, depending on the variety) and its lack of economic value [21,22,23]. Therefore, there is a significant opportunity to valorize this residue through saponin extraction, creating a sustainable product with potential applications as a biosurfactant, emulsifier, and detergent, and in agriculture for pest and disease control.

In the saponin extraction process, ultrasound technology has typically been employed [24,25,26]; however, this method faces limitations when it comes to scaling up [27,28]. Hydrodynamic cavitation presents a viable alternative, offering low manufacturing costs and high efficiency, as demonstrated in the extraction of bioactive compounds, e.g., flavonoids, phenolics, antioxidants, and pectin [27,29,30,31,32]. For the purification process, membrane technology stands out as a sustainable and scalable option. However, its use is limited to flat membranes with low working volumes. 

In this study, with the aim of valorizing quinoa residues, hydrodynamic cavitation and membrane technology were used to recover saponin. Subsequently, the techno-functional properties of the recovered saponin were evaluated, with a focus on its surface tensioactive and emulsifying properties, with the aim of assessing its potential application as an eco-friendly detergent in textiles.

## 2. Results and Discussion

### 2.1. Extraction and Characterization of Quinoa Saponin

In this work, a saponin extraction yield of 56.30% (calculated as the percentage of initial saponin in the powder recovered after the extraction process) was achieved using hydrodynamic cavitation (HC) with only water. This can be attributed to the high turbulence and physicochemical effects generated during the violent collapse of bubbles [32]. After cavitation, the solid was sedimented at pH 4.5, residual particles were removed by ultrafiltration, and the permeate was concentrated by nanofiltration. Subsequently, from the retentate, the saponin was extracted by adding methanol, and the solvent was removed, obtaining an extract free of solvent named saponin extract (SE).

The SE contained a saponin concentration of 178.68 g/L. After drying, the saponin purity was determined to be 42%. The dried extract also contained 3.98 g/L of total phenols, 1.24 g/L of reducing sugars, and 24.77 g/L of soluble proteins. These results are close to the ones previously reported by Lozano et al. [33], which included a purity of 45% (*w*/*w*), which was achieved by extracting saponins from *Chenopodium quinoa* Willd residues using a maceration method for 72 h, but with hydroalcoholic solvent (25% of EtOH), enhancing the extraction yield by increasing the ethanol concentration to 50%. However, in our study, the extraction and purification processes were not extensively evaluated, as our focus was on assessing the techno-functional properties at lower purity levels. It is worth noting that commercial products like Soapnuts Extract, manufactured by Wellgreen Technology Co. (Shaanxi, China), are available at 40% purity.

### 2.2. Determination of Critical Micelle Concentration (CMC)

**Figure 1:**
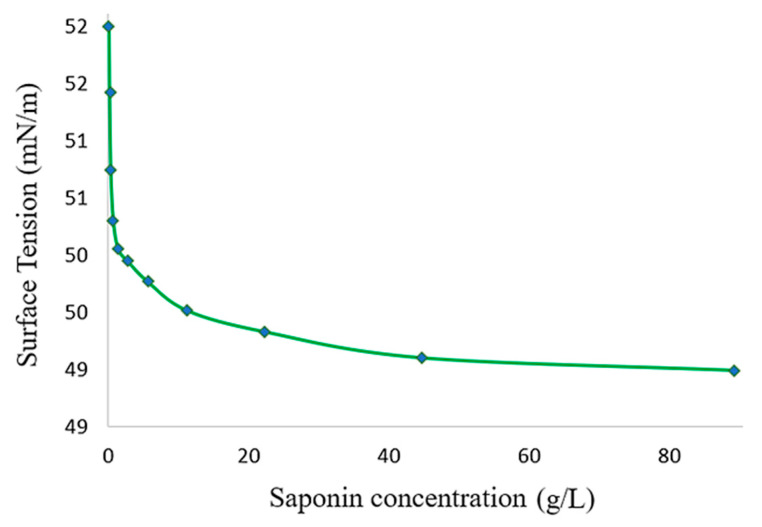
Surface tension evaluation at different saponin extract concentrations.

The CMC value of the saponin extract was 1.24 ± 0.14 g/L (Figure 1), with a corresponding surface tension of 50 mN/m, which was determined according to the method proposed by Cazals et al. [34], Ramesh et al. [35], and Springer-Verlag et al. [36]. The CMC is one of the most important physical parameters influencing a surfactant’s properties. It is defined as the specific concentration at which spontaneous micelle formation begins. Beyond the CMC, increasing the surfactant concentration does not result in further reductions in interfacial tension [37].

The CMC establishes a concentration threshold for emulsifier efficacy; surfactants with lower CMC values will solubilize hydrophobic solutes at lower surfactant concentrations compared to those with higher CMC values; therefore, applications of industrial surfactants are often based on their CMC values [38].

The CMC obtained in this study was better than the value of 2.4 g/L reported for sodium dodecyl sulfate by Verza et al. [39], and higher than the CMC reported for *Chenopodium quinoa* saponin extract, with values between 0.39 and 0.37 g/L (purity and extraction process are not available). The variation among these findings can primarily be attributed to the purity of the extract (42% in this study). This relationship is supported by Mitra and Dungan [38], who found that the CMCs of different commercial *Quillaja* saponins from Sigma Chemical Co, Acros Organics B.V.B.A. (St. Louis, MO, USA), and Penco of Lyndhurst Inc. (Lyndhurst, NJ, USA) were 0.51, 0.70, and 0.72 g/L, respectively. This indicates that a higher-purity saponin fraction corresponds to a lower CMC, whereas lower-grade saponin fractions exhibit higher CMC values [38].

Moreover, the lowest surface tension reached by the SE was 49.10 mN/m, which is higher than that of commercial surfactants such as SDS (35.6 mN/m) and Tween 80 (41.7 mN/m), as reported by Chen et al. [40]. However, in the same study, the authors achieved a reduction in water surface tension from 72 mN/m to 50.0 mN/m by employing saponins extracted from the defatted seed meal of *Camellia oleifera* with 39.5% purity (CMC: 1 g/L). These results were very similar to those achieved in this study concerning the CMC, purity of the saponin extract, and reduction in surface tension. Based on these findings, the SE studied could have potential in detergent formulation.

### 2.3. Determination of Emulsifying Index

**Figure 2:**
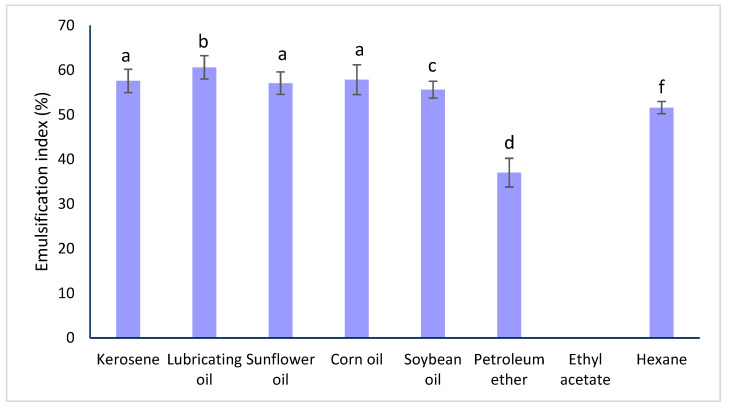
The emulsification index after 24 h. ^a,b,c,d,f^ Bars with the same letter are not significantly different (*p* > 0.05) according to a Duncan test.

The emulsifying index (EI) of the SE was evaluated in hydrophobic substrates (sunflower oil, corn oil, soybean oil) and organic solvents (kerosene, lubricating oil, petroleum ether, ethyl acetate, hexane) at a saponin concentration of 2.79 g/L, considering their good tensioactive activity at this concentration, and the results are shown in Table 1 and Figure 2.

These results indicate that the saponin extract formed efficient and stable emulsions when mixed with kerosene (EI_24_ = 57.58%), lubricating oil (EI_24_ = 60.61%), sunflower oil (EI_24_ = 57.06%), corn oil (EI_24_ = 57.86%), soybean oil (EI_24_ = 55.627%), and hexane (EI_24_ = 51.587%). In all these emulsions, the EI_24_ values were higher than 50%, which is considered stable. Additionally, a statistical study using the Multiple Range (Duncan) test revealed that there was no significant difference between the EI_24_ of SE with kerosene, sunflower oil, and corn oil compared to lubricating oil, soybean oil, petroleum ether, and hexane, revealing lubricating oil as the best organic solvent for forming emulsions with the SE at 42% purity. These results indicate the emulsifying ability of SE in its interaction with highly hydrophobic materials such as aliphatic (hexane), mixtures containing long-chain and aromatic hydrocarbons (kerosene and lubricating oil), and some vegetable oils of saturated and unsaturated C18 chains (sunflower, corn, and soybean oil). On the other hand, SE mixtures with petroleum ether and ethyl acetate resulted in EI_24_ values of 37.04% and 0%, respectively.

Additionally, the EI_24_ values in the oils were better than those obtained by Bezerra et al. [41] when evaluating the EI_24_ of *Chenopodium quinoa* saponins with coconut, sunflower, and grape oil, reaching values between 41% and 51% when evaluated at saponin concentrations of 3.3–6 g/L. The difference between these EI results could be associated with purity. For example, in the study of Sotomayor-Gerding [42], pure saponin from *Chenopodium quinoa* at 1.9 g/L was used as a natural emulsifier with canola oil, reaching an emulsion index (EI) of around 100% in the first three days.

Nevertheless, EI_72_ differed from EI_24_, and the combination of SE with lubricating oil lost stability at 72 h, decreasing to 50.43%. This instability led to kerosene, sunflower oil, corn oil, and soybean oil being identified as the best substrates for emulsification with SE. It is important to note that the EI for kerosene, sunflower oil, corn oil, and soybean oil did not show significant differences at 72 h, as confirmed by the Duncan Test. Our current work highlights the stability of emulsions using saponins from *Chenopodium quinoa* when working with vegetable oils over time, which directs our focus toward studying saponin extract for detergent applications. Therefore, they represent potential candidates for use in various industries such as petrochemicals, food, pharmaceuticals, and cosmetics.

### 2.4. Experimental Design for Optimization of Emulsifying and Tensioactive Properties

The effect of variables such as temperature, pH, and salinity on surface tension and the emulsification index were evaluated through a Box–Behnken design, and the results are shown in Table 2. As observed, the surface tension was in the range of 49.01–51.33 mN/m, reaching the lowest surface tension at the central point (60 °C, salinity of 100,000 ppm, and pH of 7), and the emulsification indexes were in the range of 30–67.5%, with highest value in run 13 (60 °C, salinity of 150,000 ppm, and pH of 12).

**Figure 3:**
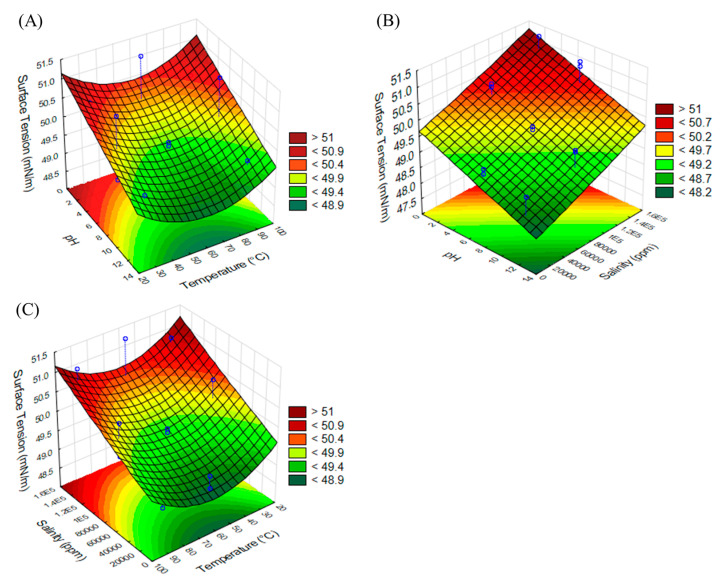
Effect of variables on surface tension. (**A**) pH vs. temperature, (**B**) pH vs. salinity, and (**C**) salinity vs. temperature. In each figure, graphic was constructed considering central point of third variable.

The statistical significance of the quadratic empirical model and the effect of each factor on surface tension were assessed by performing an ANOVA test (Table 3). The model was reduced by removing from it insignificant terms (*p* > 0.05), except if required for model hierarchy. As shown in Table 3, the model (Equation (1)) was significant at a 95% confidence level, and it had a high R-squared value of 0.94. The set of the mathematical model was confirmed by the *p*-value (<0.0001), F-value (28.85), and non-significant lack-of-fit test (*p*-value > 0.05) at a confidence level of 95%. For the surface tension, the *p*-value indicated that both pH and salinity had a significant effect on surface tension, with temperature’s effect represented by a quadratic term in the model (Figure 3).

ST (mN/m) = 50.41 + 0.004A − 0.05B + 0.00002C − 1.43 × 10^−6^ AC + 0.0004B^2^ (1)

where ST = surface tension, A = pH, B = temperature, and C = salinity.

**Figure 4:**
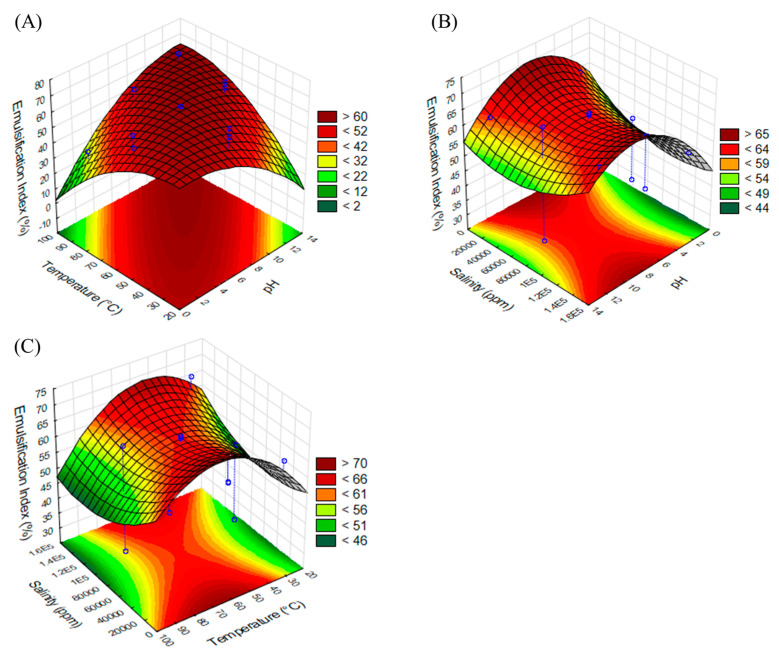
Effect variables on emulsification index obtained using saponin extract. (**A**) Temperature vs. pH, (**B**) salinity vs. pH, and (**C**) salinity vs. temperature. In each figure, graphic was constructed considering central point of third variable.

For the emulsification index, an ANOVA test was also performed, with the results presented in Table 4. As shown in the table, the model (Equation (2)) was significant at a 95% confidence level, and it had an R-squared value of 0.85. The set of the model was also confirmed by the *p*-value (0.019), F-value (5.54), and non-significant lack-of-fit test (*p*-value > 0.05) at a confidence level of 95%. In the case of the emulsification index, the *p*-values for pH, salinity, and temperature were higher than 0.05, and therefore did not have a significant effect on the emulsification index; however, the coefficient for pH (2.52) showed a direct influence on the response variable (EI_24_). Moreover, the coefficient of the variables suggests that pH had a greater influence on the emulsification index than salinity and temperature (Figure 4A–C).

EI (%)= 62.47 − 1.49A + 0.36B − 1.93 × 10^−4^ C + 0.10AB − 0.27A² − 8.74 × 10^−3^ B² (2)

where EI = emulsification index, A = pH, B = temperature, and C = salinity.

The response variables were optimized to maximize the emulsification index and to minimize the surface tension. The optimized conditions were a pH of 9.05, a temperature of 66 °C, and salinity of 10,000 ppm for NaCl. Under these conditions, the quadratic models predicted a surface tension of 48.94 ± 0.24 mN/m and an EI_24_ of 67.95 ± 5.87% (average ±95% confidence interval). The predicted values were validated experimentally, yielding a surface tension of 49.02 ± 2.45 mN/m and an EI_24_ of 63 ± 3.15%.

The obtained results are in concordance with those reported in the literature, e.g., the effect of pH on the surface tension properties of saponins from *Quillaja saponaria* showed that for all concentrations of saponin studied, and at pH values below and above their pKa (pH 3 and 7), a reduction in interfacial tension was achieved, which explains why in our study, we had better results (less surface tension) when we worked with higher pH [43]. Also, the highest reduction in CMC values from Agave sisalana saponins was reached when the values of pH were in the range of 10–11 with temperatures around 55–60 °C and a saline concentration from 20 to 60 ppm [44]. Moreover, the effect of salinity exhibited similar behavior to that reported by Nowrouzi et al. [45], who studied interfacial tension reduction using extracted saponin from the Anabasis Setifera plant. In their work, the surfactant performed effectively at different salinities of injection water, with lower salinity resulting in smaller interfacial tension.

The behavior of the EI concurs with the results reported by Sotomayor-Gerding et al. [42] when they evaluated *Quillaja saponaria* and *Chenopodium quinoa* saponins as natural emulsifiers in the formation of astaxanthin-enriched canola oil emulsions; the emulsifications obtained were stable over a wide range of pH values (4–10) but exhibited particle aggregation at lower pH and salt conditions and high temperatures. 

### 2.5. Results of the Detergency Test

**Figure 5:**
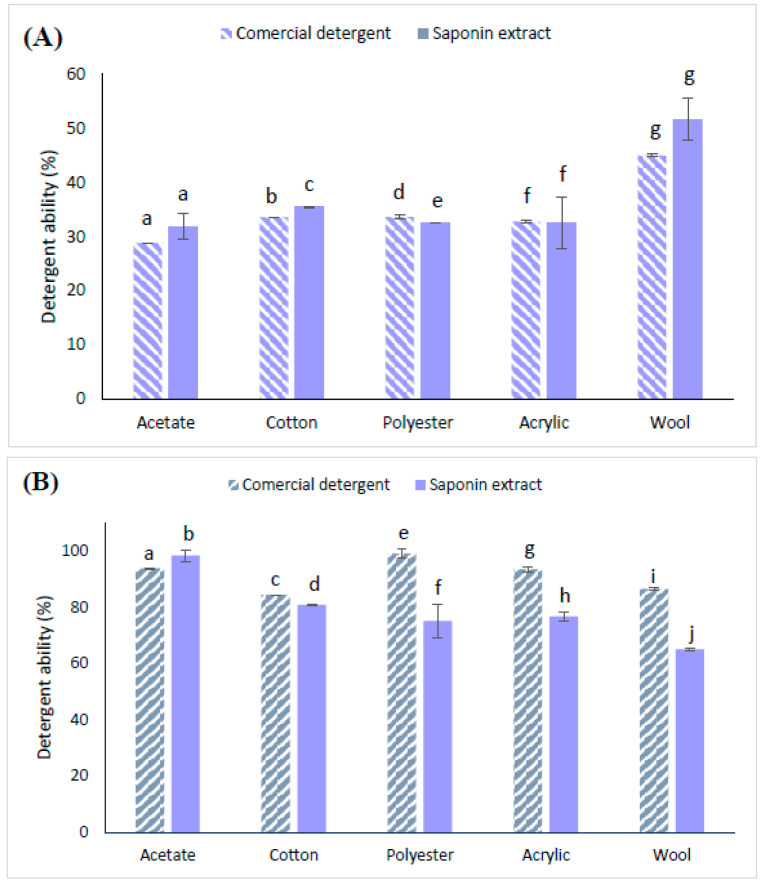
Detergency test. (**A**) Saponin extract (SE) and commercial detergent in palm oil swatches, and (**B**) SE and commercial detergent in coffee swatches. ^a–j^ Bars with the same letter are not significantly different (*p* > 0.05) according to Duncan test.

A detergency test was performed, and the results are shown in Figure 5. As seen in Figure 5 for palm oil swatches, the maximum washing performance for formulated saponin extract was reached in the wool weave (51.65 ± 3.82%), with a value not significantly different from that denoting the washing performance of commercial detergent for wool weave (44.98 ± 0.23%). Additionally, acetate and acrylic weaves did not present a significant difference between the washing performance of commercial detergent and the saponin extract either. However, for cotton and polyester weaves, there was a significant difference between the commercial detergent and the saponin extract, which showed lower washing performance, although the values were closer. The detergency reached was also similar to that observed by Yang et al. [46] for saponins from the pericarp of *Sapindus mukorossi*. The authors reported a total saponin content in crude saponin extract of 85% (*w*/*w*) and a remotion of sebum using saponin extract of 60%.

For coffee swatches, all the weaves (acetate, cotton, polyester, acrylic, and wool) presented significant differences between the commercial detergent (CD) and the saponin formulate (SED). For the detergency test in coffee swatches, the saponin extract showed a better yield in the acetate weave (98.63 ± 2.07%) than the commercial detergent (93.96 ± 0.13%), with a significant difference. For the other weaves (cotton, polyester, acrylic, and wool), the commercial detergent showed better efficiency in removing coffee stains compared to saponin extract; however, SED exhibited a good percentage of detergency (above 65.15%), indicating the potential of the saponin extract as a detergent.

## 3. Materials and Methods

### 3.1. Material and Chemicals

Quinoa (*Chenopodium quinoa*) residue powder, containing 26% saponin, was kindly provided by a local company (Arequipa, Peru). This powder was obtained through the mechanical scarification of quinoa grains, with a particular emphasis on grains with high saponin content. All other chemicals utilized were of analytical grade and sourced from Merck & Co., Inc. (New York, NY, USA).

### 3.2. Saponin Extract from Quinoa Powder

The saponin was extracted from quinoa powder residues using a hydrodynamic cavitation (HC) system with 10 L of volume work. The HC system was equipped with a centrifugal pump, valvules, a cavitation device (based on the combination of an orifice plate and a venturi tube), and a recirculation tank [47]. The experiment of extraction was performed using 800 g of powder with 7 L of water, and the operation conditions were 2 bar of inlet pressure for 15 min, with the temperature maintained below 50 °C. Following the hydrodynamic cavitation (HC) process, solid particles in the extract were precipitated at a pH of 4.5 and adjusted using 0.5 N H_2_SO_4_. The supernatant was then recovered and subjected to ultrafiltration using a ceramic crossflow filtration module obtained from Inopor^®^ (Scheßlitz, Germany), equipped with a tubular ceramic membrane with a 30 nm pore size (length of 25 cm, channel diameter of 7 mm, and outer diameter of 10 mm), to obtain a concentrated extract. This concentrated extract was mixed with methanol in a 1:1 ratio, resulting in the precipitation of the insoluble fraction. Subsequently, the liquid fraction was recovered and evaporated using a rotary evaporator, resulting in an extract with a high concentration of saponin, referred to as saponin extract (SE).

### 3.3. Analysis of Saponin Extract

The saponin extract was analyzed regarding saponin, total phenolic compounds, reducing sugars, and protein content. The saponin content was measured according to the method reported by Hiai et al. [48]. Oleanolic acid (OA) was used as a standard, the absorbance was measured at 531 nm, and the results were expressed in mg of equivalent of OA per g of powder (mg EOA/g of powder). Total phenolic compounds (TPC) were measured following the Folin–Ciocalteu assay described by Singleton and Rossi [49]. Moreover, soluble proteins and reducing sugars were measured according to the procedure reported by Lowry and Miller [50,51], respectively.

### 3.4. Surface Tension and Critical Micelle Concentration (CMC)

The surface tension (ST) of the crude saponin extract was measured at different concentrations (0.08–178.68 g/L). The analysis was performed at room temperature using a force tensiometer Krüss K20, KRÜSS Scientific (Hamburg, Germany). The critical micelle concentration (CMC) was determined by plotting the ST (mN/m) as a function of the SE concentration (g/L). All tests were performed in triplicate.

### 3.5. Emulsifying Properties of Saponin Extract

The emulsification index (EI) of the saponin extract (SE) was analyzed following the method proposed by Cooper and Goldenberg [52]. The emulsifying properties of SE, using 1 mL of SE with a saponin concentration of 2.79 g/L and 1 mL of various oils (sunflower oil, corn oil, soy oil) or organic solvents (kerosene, lubricating oil, petroleum ether, ethyl acetate, hexane). The mixtures of SE with hydrophobic substrates or organic solvents were vigorously shaken for 2 min and then allowed to stand for up to 72 h. EI measurements were conducted at 24 h (EI_24_), 48 h (EI_48_), and 72 h (EI_72_). All tests were performed in triplicate.

### 3.6. Effect of Temperature, pH, and Salinity on Emulsifying and Tensioactive Properties of Saponin Extract

The effects of temperature (30–90 °C), pH (2–12), and salinity (10–150 g/L of NaCl) on the response variables, such as the emulsification index, after 24 h (EI_24_) and the tensioactive properties (ST) were evaluated using a Box–Behnken design. All experiments were carried out using 10 mL of saponin extract containing 2.79 g/L of saponin. The minimum and maximum values for each parameter were fixed according to the previously reported studies [38,45,53,54].

The emulsifying properties were evaluated using kerosene, and the experiment was performed in triplicate according to the method described in Section 2.5. The surface tension values of the saponin extract in different conditions according to the experimental design were measured at room temperature in a force tensiometer (Kruss K20, Hamburg, Germany) as described in Section 2.4.

### 3.7. Detergency Test

The washing performance of the SE and a commercial detergent was determined via a detergency test (%) according to the procedure reported by Maurad et al. [55] and Tanthakit et al. [56]. The test was carried out using 25 mL of SE at 2.79 g/L, under pre-optimized conditions (pH 9.05 and 10,000 ppm of NaCl), with a multifiber test #10 comprising six different types of weaves (acetate, cotton, nylon, polyester, acrylic, wool) stained with substances of vegetal origin (coffee) and fat origin (palm oil) under representative washing conditions (300 rpm for 30 min).

To compare the effectiveness of SE in removing stains, controls with a commercial detergent and only water were also evaluated. Moreover, a target containing only the saponin extract with the band of weaves was prepared due to its orange color, which could affect the results obtained. For the commercial detergent, a new band of weaves was analyzed as its target.

Detergency performance was calculated using Equation (3) and it was determined by the reflectance measurement of the samples in a colorimeter (Colormate, Scinco Co., Ltd., São Paulo, Brazil).

D (%) = [(A − B) (C_0_ − B)] × 100 (3)

where C_0_ = the reflectance measurements of the unsoiled swatches, A = the post-wash soiled swatches’ reflectance, B = the pre-wash soiled swatches’ reflectance, and D = detergency performance.

### 3.8. AI-Assisted Tool

During the preparation of this work, the authors used ChatGPT 4.0 (https://chatgpt.com/) in order to improve only the readability and language. After using this tool, the authors reviewed and edited the content as needed and take full responsibility for the content of the publication.

## 4. Conclusions

The present study evaluated the tensioactive and emulsification properties of saponin extract obtained from residues of a milling process of *Chenopodium quinoa* before human consumption. Both properties were successfully assessed using a Box–Behnken design (optimized conditions of 66 °C, 10,000 ppm of NaCl, and pH 9.05) and then proven as an effective detergent in the removal of vegetable and fat stains in different weaves, achieving a good percentage of detergency closer or even higher than that reached by a commercial detergent. The results reported here are promising because they show the viability of saponin extract as a biosurfactant and the potential industrial applications of these bio-based products.

## Figures and Tables

**Table 1:** Emulsification index of saponin extract after 24, 48, and 72 h with different substrates.

Substrate	Emulsification Index After Different Times (h)		
	24	48	72
Kerosene	57.58 ± 2.62 ^a^	54.88 ± 0.58 ^a^	53.79 ± 1.31 ^a^
Lubricating oil	60.61 ± 2.62 ^b^	52.46 ± 2.50 ^b^	50.43 ± 0.74 ^b^
Sunflower oil	57.07 ± 2.52 ^a^	55.48 ± 1.85 ^c^	53.12 ± 2.41 ^a^
Corn oil	57.86 ± 3.32 ^a^	54.25 ± 1.74 ^a^	53.49 ± 2.33 ^a^
Soybean oil	55.63 ± 1.91 ^c^	54.32 ± 0.59 ^a^	53.22 ± 1.54 ^a^
Petroleum ether	37.04 ± 3.21 ^d^	53.82 ± 1.25 ^a^	37.52 ± 1.18 ^c^
Ethyl acetate	0.00 ± 0.00 ^e^	0.00 ± 0.00 ^d^	0.00 ± 0.00 ^d^
Hexane	51.59 ± 1.36 ^f^	49.07 ± 1.60 ^e^	58.75 ± 0.72 ^e^

The reported value corresponds to the average of triplicate experiments ± standard deviation (SD). ^a,b,c,d,e,f^ Means within a column with the same letter are not significantly different (*p* > 0.05) according to a Duncan test.

**Table 2:** Surface tension and emulsification index obtained in experimental runs.

Run	Experimental Variables			Response Variables	
	pH	Temperature (°C)	Salinity (ppm)	Surface Tension (mN/m)	Emulsification Index (%)
1	12	90	80,000	49.57	67.50
2	2	90	80,000	50.42	34.00
3	7	30	10,000	49.22	61.00
4	7	60	80,000	49.49	63.50
5	7	90	150,000	51.04	49.50
6	7	30	150,000	50.9	66.30
7	2	60	150,000	51.33	53.10
8	7	60	80,000	49.66	64.00
9	7	60	80,000	49.76	59.00
10	7	90	10,000	49.08	58.70
11	2	30	80,000	50.52	54.70
12	2	60	10,000	49.33	61.00
13	12	60	150,000	49.01	64.70
14	12	30	80,000	49.61	30.00
15	12	60	10,000	49.01	60.80

**Table 3:** ANOVA for surface tension.

Source	Sum of Squares	Df	Mean Square	F-Value	*p*-Value	
Model	7.96	5	1.59	28.85	<0.0001	Significant
A—pH	2.42	1	2.42	43.83	<0.0001	
B—Temperature	0.01	1	0.01	0.04	0.838	
C—Salinity	3.98	1	3.98	72.02	<0.0001	
AC	1	1	1	18.11	0.002	
B²	0.57	1	0.57	10.25	0.011	
Residual	0.50	9	0.06			
Lack of Fit	0.46	7	0.07	3.52	0.239	not significant
Pure Error	0.04	2	0.02			

**Table 4:** ANOVA for emulsification index (EI_24_).

Source	Sum of Squares	Df	Mean Square	F-Value	*p*-Value	
Model	1525.75	9	169.53	4.93	0.0469	significant
A—pH	51	1	51	1.48	0.2778	
B—Temperature	0.66	1	0.66	0.019	0.8952	
C—Salinity	7.8	1	7.8	0.23	0.6541	
AB	846.81	1	846.81	24.61	0.0042	
A²	172.62	1	172.62	5.02	0.0752	
B²	228.25	1	228.25	6.63	0.0497	
C²	111.19	1	111.19	3.23	0.1322	
Residual	172.07	5	34.41			
Lack of Fit	161.95	3	53.98	10.66	0.087	not significant
Pure Error	10.13	2	5.06			

## Data Availability

The findings of this research are supported by data from the corresponding author, K.A.G.B., which are available upon reasonable request.
